# A randomized, crossover comparison of ketamine and electroconvulsive therapy for treatment of major depressive episodes: a Canadian biomarker integration network in depression (CAN-BIND) study protocol

**DOI:** 10.1186/s12888-020-02672-3

**Published:** 2020-06-02

**Authors:** Jennifer L. Phillips, Natalia Jaworska, Elizabeth Kamler, Venkat Bhat, Jean Blier, Jane A. Foster, Stefanie Hassel, Keith Ho, Lisa McMurray, Roumen Milev, Zahra Moazamigoudarzi, Franca M. Placenza, Stéphane Richard-Devantoy, Susan Rotzinger, Gustavo Turecki, Gustavo H. Vazquez, Sidney H. Kennedy, Pierre Blier, on behalf of the CAN-BIND Investigator Team

**Affiliations:** 1 The Royal’s Institute of Mental Health Research, 1145 Carling Avenue, Ottawa, ON K1Z 7K4 Canada; 2 Department of Psychiatry, University of Ottawa, 1145 Carling Avenue, Ottawa, ON K1Z 7K4 Canada; 3 Department of Cellular and Molecular Medicine, University of Ottawa, 451 Smyth Road, Ottawa, ON K1H 8M5 Canada; 4 University Health Network, 399 Bathurst Street, Toronto, ON M5T 2S8 Canada; 5 Unity Health Toronto, St. Michael’s Hospital, 193 Yonge Street, 6th floor, Toronto, ON M5B 1M4 Canada; 6 Department of Psychiatry, University of Toronto, 250 College Street, 8th floor, Toronto, ON M5T 1R8 Canada; 7 Montfort Hospital, 713 Montreal Rd, Ottawa, ON K1K 0T2 Canada; 8 McMaster University, and St. Joseph’s Healthcare, Hamilton, 1280 Main Street West, Hamilton, ON L8S4L8 Canada; 9 Department of Psychiatry and Mathison Centre for Mental Health Research and Education, Cumming School of Medicine, University of Calgary, 3330 Hospital Dr NW, Calgary, AB T2N 4N1 Canada; 10 Royal Ottawa Mental Health Centre, 1145 Carling Avenue, Ottawa, ON K1Z 7K4 Canada; 11 Department of Psychiatry, Queen’s University, Providence Care Hospital, 752 King Street West, Postal Bag 603, Kingston, ON K7L 7X3 Canada; 12 Department of Psychology, Queens University, 62 Arch Street, Kingston, ON K7L 3N6 Canada; 13 McGill University, 845 Rue Sherbrooke O, Montréal, QC H3A 0G4 Canada; 14 Douglas Mental Health University Institute Frank B. Common (FBC) F-3145, 6875 LaSalle Boulevard, Montréal, QC H4H 1R3 Canada

**Keywords:** Major depressive disorder, Bipolar disorder, Depression, Intravenous ketamine, Electroconvulsive therapy, Biomarkers, Neuroimaging, Genomics, Clinical trial

## Abstract

**Background:**

Recent evidence underscores the utility of rapid-acting antidepressant interventions, such as ketamine, in alleviating symptoms of major depressive episodes (MDE). However, to date, there have been limited head-to-head comparisons of intravenous (IV) ketamine infusions with other antidepressant treatment strategies in large randomized trials. This study protocol describes an ongoing multi-centre, prospective, randomized, crossover, non-inferiority trial comparing acute treatment of individuals meeting diagnostic criteria for a major depressive episode (MDE) with ketamine and electroconvulsive therapy (ECT) on efficacy, speed of therapeutic effects, side effects, and health care resource utilization. A secondary aim is to compare a 6-month maintenance strategy for ketamine responders to standard of care ECT maintenance. Finally, through the measurement of clinical, cognitive, neuroimaging, and molecular markers we aim to establish predictors and moderators of treatment response as well as treatment-elicited effects on these outcomes.

**Methods:**

Across four participating Canadian institutions, 240 patients with major depressive disorder or bipolar disorder experiencing a MDE are randomized (1:1) to a course of ECT or racemic IV ketamine (0.5 mg/kg) administered 3 times/week for 3 or 4 weeks. Non-responders (< 50% improvement in Montgomery-Åsberg Depression Rating Scale [MADRS] scores) crossover to receive the alternate treatment. Responders during the randomization or crossover phases then enter the 6-month maintenance phase during which time they receive clinical assessments at identical intervals regardless of treatment arm. ECT maintenance follows standard of care while ketamine maintenance involves: weekly infusions for 1 month, then bi-weekly infusions for 2 months, and finally monthly infusions for 3 months (returning to bi-weekly in case of relapse). The primary outcome measure is change in MADRS scores after randomized treatment as assessed by raters blind to treatment modality.

**Discussion:**

This multi-centre study will help identify molecular, imaging, and clinical characteristics of patients with treatment-resistant and/or severe MDEs who would benefit most from either type of therapeutic strategy. In addition to informing clinical practice and influencing health care delivery, this trial will add to the robust platform and database of CAN-BIND studies for future research and biomarker discovery.

**Trial registration:**

ClinicalTrials.gov identifier NCT03674671. Registered September 17, 2018.

## Background

Worldwide, major depressive disorder (MDD) carries the largest burden of disease among psychiatric, neurological, and substance-use disorders, according to the World Health Organization [1]. Adding to this burden is bipolar disorder (BP), in which patients spend most of their symptomatic time in a major depressive episode (MDE) rather than in a hypomanic or manic episode [2, 3]. Although there are various effective treatments for MDEs, a large proportion of patients do not achieve remission even after several medication trials, and significant responses usually occur after a delay of a few weeks [4]. Thus, there remain major unmet needs for treating MDEs, including higher response rates and treatments that elicit faster antidepressant effects.

Ketamine, a primarily glutamatergic *n*-methyl-D-aspartate (NMDA) receptor antagonist, appears poised to address these needs. The discovery of the rapid antidepressant effects of subanaesthetic doses of intravenous (IV) ketamine [5] has been described as one of the most important breakthroughs in the field of depression in the past 50 years [6]. Randomized, placebo-controlled trials in unipolar and bipolar depression indicate that the antidepressant response to single ketamine infusions often manifests within a few hours, is generally maximal after 24 h, and yet dissipates within 7 days [7, 8]. While the effects of a single infusion are transient, repeated infusions have sustained antidepressant effects [9]. Moreover, our group and others have reported cumulative antidepressant effects with repeated infusions that lead to increased patient response rates over time [10–12], with further prolongation with once-weekly maintenance infusions in previously treatment-resistant depression [10]. Overall, ketamine appears to yield higher response rates than other pharmacological treatment strategies for depression; however, only a few randomized controlled trials have directly compared ketamine with other antidepressant interventions [13, 14].

Electroconvulsive therapy (ECT) remains the gold standard in treating severe and/or treatment-resistant MDEs [15]. Response rates to ECT are high (50–80%), and latency to response is shorter than for classical pharmacotherapy [16–18]. Despite these benefits, access to ECT is limited and lengthy wait-lists are common. Moreover, its high costs, potential for side effects, including cognitive deficits, and enduring stigma have resulted in underutilization of ECT as a treatment for MDEs.

One of the current goals in psychiatry is the identification of biological phenotypes (or biomarkers) that could improve characterization of the likelihood that a patient will respond to a specific treatment strategy. This is particularly pertinent in context of ECT or ketamine treatment, as both are currently reserved for the most treatment resistant and severely depressed patients. Candidate biomarkers for outcome prediction in depression include structural and functional neuroimaging profiles, as well as genetic, epigenetic, proteomic, and metabolic features [19]. Currently, it is unclear how baseline biomarker measures moderate clinical outcomes. Exploring how these measures are impacted by ECT or ketamine may further our understanding of the underlying mechanisms of action of these interventions in alleviating depressive symptoms.

The primary objective of this longitudinal, multi-centre non-inferiority trial is to compare acute treatment of MDEs with repeated IV ketamine and ECT in terms of efficacy, speed of therapeutic effects, side effect profiles, and health care resource utilization. A secondary objective is to assess whether ketamine response can be sustained with a gradual spacing of ketamine maintenance infusions, as is the case for ECT. Finally, through the collection of clinical, cognitive, neuroimaging and molecular data according to Canadian Biomarker Integration Network in Depression (CAN-BIND) protocols [20, 21], we aim to identify biomarkers that predict or moderate ketamine or ECT response.

## Methods/design

### Protocol overview

**Fig. 1:**
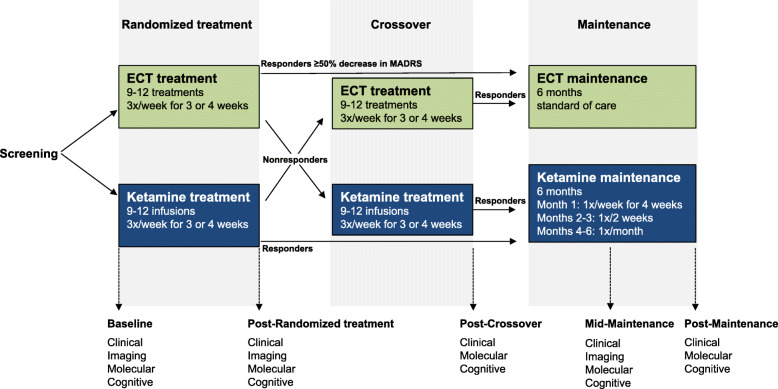
Study design

The study design is a multi-centre, prospective, longitudinal, randomized, crossover, non-inferiority trial comparing ECT and IV ketamine infusions (Fig. 1). After being referred for and consenting to ECT, individuals with MDD or BP currently experiencing a MDE are then offered the opportunity to enroll in this study. Participants who consent are randomized to ECT or ketamine by the site coordinator using a computerized randomization sequence stratified by centre with a 1:1 allocation using random block sizes. During the randomized treatment phase, participants receive a course of thrice-weekly ECT or ketamine infusions for 3 or 4 weeks. Non-responders to the first randomized treatment (< 50% reduction in depression severity as assessed by blinded raters using the Montgomery-Åsberg Depression Rating Scale [MADRS] [22], and MADRS score ≥ 22) crossover to receive treatment with the second strategy. Participants continue taking any concomitant medication throughout the study, with no change in medication regimen permitted during the initial randomization or crossover phase. Individuals who achieve response (≥ 50% reduction in MADRS scores and MADRS score < 22) in either the randomized treatment or crossover phase are eligible to enter the maintenance phase, while treatment non-responders following the crossover phase exit the study. During the 6-month maintenance phase, ECT is administered at the discretion of the prescribing physician as per standard of care, while ketamine is administered according to a set schedule. Clinical, cognitive and molecular assessments are conducted at baseline (prior to treatment initiation), post-randomized treatment phase (following weeks 3 or 4), post-crossover phase (if present), and mid- and post-maintenance phase. Neuroimaging occurs at baseline, post-randomized treatment phase, and mid-maintenance (imaging occurs less frequently due to cost and increased participant burden). As part of the CAN-BIND Integrated Discovery Program, this trial is principally funded by the Ontario Brain Institute (OBI). The lead site overseeing the clinical trial is the Royal’s Institute of Mental Health Research (IMHR), affiliated with the University of Ottawa and the Principal Investigator is P. Blier.

### Participants

A total of 240 participants are being recruited at four centres in Canada: Ottawa (Royal Ottawa Mental Health Centre), Toronto (University Health Network), Kingston (Providence Care Hospital), and Montreal (Douglas Mental Health University Institute). The research ethics boards at each site have approved the study protocol. Participants are being recruited directly from ECT service waitlists and referrals at each participating hospital. Enrollment is expected to last 3 years from the first patient enrolled. Table 1 lists the inclusion and exclusion criteria for participants.

**Table 1:** Inclusion and exclusion criteria for participants

*Inclusion criteria*	
• Ability to freely provide written informed consent before initiation of study related procedures.	
• Inpatients or outpatients referred to and eligible for standard ECT. Willing to accept randomization to either ECT or IV ketamine arms.	
• Males and females between 18 and 70 years of age.	
• Meet Diagnostic and Statistical Manual for Mental Disorders, fifth edition (DSM-5) [23] criteria for MDD or BP without psychotic symptoms as confirmed by the MINI International Neuropsychiatric Interview (MINI) [24].	
• Currently in a MDE confirmed by the MINI.	
• Montgomery-Åsberg Depression Rating Scale (MADRS) [22] total score of ≥26 at screening and at randomization, with no more than 20% improvement between these two visits.	
• Body mass index < 35.	
• Montreal Cognitive Assessment (MOCA) [25] score ≥ 24.	
• Ability to understand and comply with study requirements, as judged by the investigator(s).	
*Exclusion criteria*	
• Depression secondary to stroke, cancer or other medical illnesses.	
• Prior or current substance abuse or dependence (except for caffeine or nicotine dependence) and/or recent history (last 12 months) of current alcohol abuse or dependence, as defined by DSM-5 criteria [23].	
• A positive toxicology screen for drugs that are not prescribed.	
• Pregnant, breastfeeding, or of childbearing potential and not willing to use an approved method of contraception during the study.	
• Evidence of clinically relevant disease or unstable medical illness.	
• Clinically significant deviation from reference ranges in clinical laboratory tests.	
• Clinically significant electrocardiogram results as judged by the investigator(s) or cardiologist.	
• History of seizure disorder, except febrile convulsions.	
• Known history of intolerance or hypersensitivity to ketamine.	

### Procedure

Individuals first provide signed consent to receive ECT as part of their care. Subsequently, at the screening visit, all participants provide written informed consent in adherence with Good Clinical Practice guidelines before initiation of any study related procedures. Participants then undergo a full medical work-up to ensure ECT and ketamine treatment suitability, which includes a medical history, physical examination*,* height, weight and vital signs measurements, 12-lead electrocardiogram, urinalysis, standard clinical laboratory tests, toxicology and pregnancy screenings, as well as recording of concomitant medication regimens. Participants are also assessed for contraindications to magnetic resonance imaging (MRI). They then undergo a psychiatric interview to confirm a diagnosis of MDD or BP according to Diagnostic and Statistical Manual for Mental Disorders, fifth edition (DSM-5) [23] criteria using the MINI International Neuropsychiatric Interview (MINI) Version 7.0.2 [24]. Depressive symptom severity is assessed using the MADRS, while lifetime history of suicidal ideation, behaviours, and attempts are assessed using the Columbia-Suicide Severity Rating Scale (C-SSRS) [26]. Absence of cognitive impairment is ascertained with the Montreal Cognitive Assessment (MOCA) [25]. An up-to-date Antidepressant Treatment History Form (ATHF) [27] is used to assess pharmacological treatment history (type, dose, duration). Participants provide sociodemographic information using standardized self-report forms and complete various self-report questionnaires assessing their pre-treatment psychiatric symptoms and quality of life.

Following the screening visit, eligible participants are formally enrolled in the study, randomized, and undergo baseline data collection, including a computerized cognitive battery, an MRI scan, and blood draws for genomic and molecular markers.

### Treatments

For both arms, whether treatment is initiated on an inpatient or outpatient basis depends on clinician judgment and logistical constraints at each participating site. ECT is administered according to standard of care with selection of specific treatment parameters (i.e., unilateral or bilateral electrode placement, and pulse duration) at the discretion of the treating psychiatrist. Each course of ECT generally starts with unilateral administration. According to patient response, physicians may opt to increase to bitemporal or bifrontal treatments. Selection of anaesthetic agents and muscle relaxants are at the discretion of the anaesthesiologist, with the exception of ketamine, which is not to be used as an anaesthetic agent during ECT.

Racemic ketamine hydrochloride is administered at a dose of 0.5 mg/kg diluted in 0.9% saline, over a 40-min period by an IV pump. Infusions are administered in ECT or post-anaesthetic care recovery rooms at each of the participating sites by study nurses or anaesthesiologist assistants under the supervision of a trained physician and with constant cardiorespiratory monitoring. Vital signs are monitored at 5-min intervals throughout each infusion and afterward until the return of preinfusion levels. Participants receiving ketamine are required to abstain from consuming grapefruit juice (a potent 3A4 cytochrome inhibitor that may slow ketamine elimination) on infusion days [28]. All participants must avoid taking anticonvulsants and benzodiazepines from the day preceding treatments as they attenuate ketamine response [29] and increase the seizure threshold, factors that can reduce the efficacy of ketamine and ECT.

At each treatment visit, clinical providers assess treatment response, side effects, adverse events and suitability for continued treatment. All reasonable efforts will be made to retain participants in the study without exerting undue pressure. Interventions may be discontinued based on clinical judgement in accordance with the risk-benefit ratio. Side effects are recorded using the clinician-rated Toronto Side Effect Scale (TSES) [30] and an adverse event log. A Data and Safety Monitoring Board (DSMB) has been assembled to monitor participant safety and treatment efficacy during the trial. This panel is comprised of three study independent experts: a psychiatrist, an anesthesiologist, and a statistician. Healthcare resource use occurring outside of the study, such as inpatient hospitalization, emergency room visits or outpatient consultations, is also documented at each visit. Finally, the estimated financial cost of administering each treatment is being calculated at each site.

### Treatment schedule

#### Randomized treatment phase

For participants receiving ECT, the number of treatments (9 or 12) is determined by the treating physician. For the ketamine arm, the number of treatments is determined by participants’ antidepressant response as assessed using the MADRS. Participants achieving remission (MADRS ≤10) after 9 ketamine infusions exit the randomized treatment phase and are eligible to enter the maintenance phase (Fig. 1). Participants receiving ketamine who achieve an antidepressant response but not remission after 9 treatments receive an additional 3 infusions (12 treatments total). In both treatment arms, non-responders (< 50% MADRS decrease or a score ≥ 22) after 3 weeks may crossover to receive a full course of treatment with the alternative strategy. Responders after 12 treatments may move on to the maintenance phase.

#### Crossover phase

Participants only enter the crossover phase if they do not meet antidepressant response criteria at the end of the randomized treatment phase. During the crossover phase, ECT non-responders receive a course of 9 or 12 ketamine infusions, while ketamine non-responders undergo 9 or 12 ECT sessions. Non-responders at the end of the crossover phase exit the study. Those meeting the response criteria after the crossover phase (≥ 50% MADRS score decrease relative to pre-crossover score and MADRS score < 22) may enter the maintenance phase.

#### Maintenance phase

Treatment during the maintenance phase lasts 6 months from entry (immediately after completion of the randomized treatment or crossover phase), with a follow-up assessment at month 7. ECT maintenance follows standard of care wherein the treating physician will determine if the participant will receive ECT maintenance, and if so, the frequency of these treatments in accordance with patients’ needs and Canadian Network for Mood and Anxiety Treatments (CANMAT) 2016 Clinical Guidelines [15]. Participants undergoing ketamine maintenance treatment receive infusions according to the following schedule: 1 infusion per week for the first 4 weeks, followed by 1 infusion every 2 weeks for 2 months, and then 1 infusion every month for 3 months. Individuals who experience a relapse of depressive symptoms (MADRS ≥22 or loss of 50% improvement from baseline) with monthly ketamine infusions may return to bi-weekly infusions for the remainder of the maintenance phase. Participants who fail to meet response criteria at any two consecutive study visits during the maintenance phase discontinue treatments (if present) and exit the study. At the conclusion of study participation, all participants have a final follow-up visit and are discharged to their referring physician with treatment recommendations.

### Assessments

#### Clinical platform

Participants in both arms are clinically assessed on an identical schedule. Clinical raters receive standardized training in the administration of measures of interest, and are assessed for inter-rater reliability. They remain blind to treatment arm and are not otherwise involved in the study, while participants are instructed not to disclose their treatment arm during ratings. On visits consisting of both clinical scales and treatment, clinical ratings are administered prior to treatment. Clinical scales are listed in Table 2.

**Table 2:** Clinical assessments

*Clinician-Administered Assessments*	
Mini International Neuropsychiatric Interview (MINI)	
Montgomery-Åsberg Depression Rating Scale (MADRS)	
Clinical Global Impression (CGI)	
Young Mania Rating Scale (YMRS)	
Columbia-Suicide Severity Rating Scale (C-SSRS)	
Toronto Side Effects Scale (TSES)	
Montreal Cognitive Assessment (MOCA)	
CNS Vital Signs (CNS-VS), Computerized neurocognitive battery	
*Self-Report Assessments*	
Patient Health Questionnaire (PHQ-9)	
Generalized Anxiety Disorder 7-item scale (GAD-7)	
Quality of Life, Enjoyment and Satisfaction Questionnaire-short form (Q-LESQ-SF)	
Dimensional Anhedonia Rating Scale (DARS)	
Medical Outcome Study - short Form 36 (SF-36)	
Drug Abuse Screening Test (DAST-10)	
Beck Scale for Suicide Ideation (BSS)	

The primary study outcome measure is response rates to randomized treatment determined through percent change in MADRS scores from baseline (pre-randomized treatment phase) to end of randomized treatment. The MADRS is a 10-item scale designed to measure depression severity administered by a blinded rater using the Structured Interview Guide for the Montgomery-Åsberg Depression Rating Scale (SIGMA) [31]. Additional assessments include the Clinical Global Impression, Severity and Improvement Scales (CGI-S and CGI-I) [32], the C-SSRS, and the Young Mania Rating Scale (YMRS) [33]. Clinical assessments are obtained prior to each treatment during the first week (i.e., thrice-weekly), and then at weekly intervals throughout the randomized treatment and crossover phases. When administration of these scales occurs at shorter time intervals than the validated measurement timeframe, symptoms are assessed for the time period since last treatment.

Participants complete electronic self-report measures on the same schedule as the above-listed clinical assessments. These include the Patient Health Questionnaire (PHQ-9) [34], Beck Scale for Suicide Ideation (BSS) [35], General Anxiety Disorder 7-item Scale (GAD-7) [36], Quality of Life, Enjoyment and Satisfaction Questionnaire - Short Form (Q-LESQ-SF) [37], Dimensional Anhedonia Rating Scale (DARS) [38], and Medical Outcome Study - Short Form 36 (SF-36) [39].

#### Cognitive assessment

A computerized neurocognitive test battery is administered prior to treatment initiation and at the completion of each treatment phase (Fig. 1). CNS Vital Signs [40] is a battery comprised of well-established cognitive tests including verbal and visual memory, finger tapping, symbol digit coding, Stroop Test, a test of shifting attention, and continuous performance test. Cognitive testing is being used to assess and compare potential effects of repeated ECT and ketamine treatment on cognitive function, and as a possible marker of response prediction.

#### Neuroimaging platform

Structural and functional neuroimaging data are acquired at each participating site using a 3 T MRI scanner. Among the four sites, the following scanners are used: Siemens Biograph mMR PET/MR (Ottawa), 3 T Siemens Trio (Kingston) and Siemens Prisma 3 T (Toronto and Montreal). Extensive initial and ongoing standardization and quality control procedures have been implemented to ensure that comparable and high-quality data are acquired across sites [41]. Specifically, all sites are using either identical or comparable scanning protocols, identical participant instructions and consistent standard operating procedures (SOPs) in keeping with existing CAN-BIND imaging protocols [42]. Each site carries out quarterly scanning using the fBIRN phantom, a spherical agar phantom developed by the ‘functional Brain Imaging Research Network’ consortium [43].

Each participant undergoes 2 or 3 imaging sessions: 1) at baseline, prior to treatment initiation, 2) after 3 or 4 weeks of randomized treatment, and 3) for responders, at the mid-point of maintenance (after approximately 4 consecutive months of treatment). During each session, structural MRI, resting-state functional MRI (rs-fMRI), and diffusion tensor imaging (DTI) data are acquired, consistent with established CAN-BIND imaging protocols [42]. In brief, a T1 scan is first acquired (TR = 1760 ms, TE = 2.19 ms, slice thickness = 1 mm, FOV = 256 mm, flip angle = 15, sagittal acquisition, GRAPPA on); a T2-weighted BOLD EPI scan is then obtained while participants look at a fixation cross (TR = 2500 ms, TE = 30 ms, slice thickness = 3 mm, FOV = 210 mm, flip angle = 83, GRAPPA on, acceleration factor PE = 2, acquisition: interleaved). This is followed by DTI acquisition (TR = 8000 ms, TE = 94 ms, GRAPPA: on, acceleration factor PE = 2, 30 directions at 2 b-values:1 and 1000 s/mm^2^; monopolar diffusion, voxel size: 2.5mm^3^, 58 slices, orientation: transverse, FOV = 240 mm). An additional multi-echo magnetization prepared-rapid gradient echo (ME-MPRAGE) sequence has been added to the CAN-BIND imaging protocol across most sites for higher resolution structural images (TR = 1760 ms, TE1/2/3/4 = 1.69/3.55/5.41/7.27 ms, slice thickness = 1 mm, FOV = 256 mm, flip angle = 7, sagittal acquisition, GRAPPA on, acceleration factor PE = 2). Finally, at the Ottawa and Montreal sites, proton magnetic resonance spectroscopy (^1^H-MRS) data are acquired from the dorsal anterior cingulate cortex to measure glutamate + glutamine (Glx) (Point RESolved Spectroscopy [PRESS]; TR = 2000 ms, TE = 30 ms; voxel, water suppression BW: 70 Hz, averages: 128, 40 × 20 × 15 mm). Neuroimaging is being conducted to identify potential predictors of response and non-response, and examine changes in brain features with acute and longer-term treatment.

#### Molecular platform

Blood samples for biomarker analyses are collected at baseline and at the completion of each treatment phase (Fig. 1). Across sites, SOPs for sample preparation, transfer, receipt, and analysis, as well as material transfer agreements are established. De-identified biological samples are transferred to the Douglas Mental Health University Institute biobank (see [21] for details). In brief, transcriptomics (mRNA and non-coding RNA), DNA methylation and inflammatory cytokines will be examined to identify potential predictive markers of treatment response and changes with ECT and ketamine treatment [21]. Recently published preclinical data have indicated that the 2R,6R-hydroxy-norketamine metabolite of ketamine may be responsible for the rapid antidepressant effects observed with the racemic ketamine mixture [44]. As such, plasma levels of the stereoisomers of ketamine and its metabolites are obtained 2 h after the first infusion (ketamine has a 3 h half-life). This component is an addition to the CAN-BIND molecular platform.

### Data management

As part of the CAN-BIND program, each site has entered into a standardized Participation Agreement with OBI to facilitate transfer of data using multiple bioinformatics approaches [21]. Specific data collection and aggregation platforms are implemented through Centre for Ontario Data Exploration (Brain-CODE, https://www.braincode.ca/) Brain-CODE is a large-scale database and informatics platform developed and maintained by OBI. Brain-CODE is hosted at the Centre for Advanced Computing at Queen’s University in Kingston, Ontario. The centre is a member of the Compute Canada high-performance computing consortium, which supports regulatory-complaint processes for securing the privacy of health care data and provides expansive computing resources for complex data analysis (https://cac.queensu.ca/overview). Data-collection platforms involve REDCap (Research Electronic Data Capture) for clinical and demographic data [45], SPReD (Stroke Patient Recovery Research Database) for neuroimaging data [46], and LabKey [47] for managing molecular workflows, experiments, and raw data.

### Data analyses

The primary outcome measure is the rate of antidepressant response (i.e., percentage of participants meeting antidepressant response criteria [≥ 50% reduction in MADRS scores from baseline and MADRS score < 22]) in each arm at the end of the randomized treatment phase. A total of 240 participants will be enrolled into the randomized treatment phase across the four recruiting sites. This will allow for a 20% drop-out rate (i.e., withdrawal of approximately 40 participants), resulting in a total sample of *N* = 200. For the primary efficacy analyses, a non-inferiority margin of 20% was selected based on clinical judgment. Thus, a sample of 200 participants (*N* = 100/treatment arm) is required to be 90% certain that the upper limit of a one-sided 95% confidence interval will exclude a difference in favour of the ECT group of more than 20%. Secondary efficacy outcomes will include percent change in MADRS scores from baseline to the end of the randomized treatment phase, and remission rates (MADRS score ≤ 10). Response and remission rates will also be assessed for the crossover phase. Clinical scores will be analyzed using linear mixed models (appropriate covariates, such as site, will be included, as needed). Categorical outcomes (response/remission) will be examined using χ^2^ tests. The alpha will be set at 0.05.

Speed of therapeutic effects is defined as the number of treatments required for participants to first meet antidepressant response criteria. A Kaplan-Meier survival analysis will be used to compare the mean number of required treatments for first response to ECT and ketamine during the randomized treatment phase. Side effect burden will be quantified by summing the total frequency and severity scores of the TSES administered after the final treatment during the randomized treatment phase. Mean total TSES scores for ECT and ketamine will be compared using an ANOVA (appropriate covariates will be included). Further, side effect profiles for each treatment will be derived by identifying the most common side effects reported in each arm of the trial (present in > 5% of participants). Treatment tolerability will be estimated via dropout rates. Finally, data from CNS Vital Signs will permit comparison of any cognitive side effects associated with ECT or ketamine (compared at the end of the randomized treatment phase). Level of health care utilization (estimated using average cost of treatment administration, number of days of inpatient hospitalization, and frequency of outpatient consultations) associated with ECT and ketamine will be compared using ANOVAs.

The main outcome in the maintenance phase will be time to relapse. Due to differences in the administration schedules of ECT and ketamine during maintenance, two parallel descriptive statistics will be outlined. For each treatment, mean time to relapse (loss of antidepressant response) will be estimated using Kaplan-Meier survival analysis.

While data from each analytical platform will be analyzed independently using the methods described herein and elsewhere [21], integration of data collected across platforms will also be carried out, and will be overseen by the CAN-BIND Data Science Advisory Team comprised of Principal Investigators, domain-specific informatics advisors, and operations support. In addition to conventional statistical approaches, machine-learning approaches will be applied particularly with regards to outcome prediction (i.e., response/non-response to ECT and ketamine at 3 or 4 weeks of treatment).

## Discussion

There is a need to improve treatment outcomes among individuals with mood disorders who have failed to respond to various strategies. While ECT remains one of the most effective interventions for treatment-resistant MDEs, it is underutilized. Ketamine represents a promising new alternative treatment for depression with rapid antidepressant effects, high response rates, and fewer side effects. However, to date, ECT and ketamine have not been directly compared in a large randomized trial. Despite ketamine’s potential as an antidepressant intervention, the transient nature of its effects and frequent relapse of depressive symptoms that follow the cessation of infusions remain ongoing challenges [48]. This innovative trial aims to address these issues. First, it features a large-scale, multi-centre, head-to-head comparison of repeated ketamine infusions and ECT as treatment strategies for patients with mood disorders. Second, the trial includes a novel maintenance strategy for IV ketamine administration that involves sequentially decreasing the frequency of infusions following response. This strategy was shown to effectively maintain antidepressant response and alleviation of suicidal ideation over short-term follow-up by our group [10, 49]. Further support for this approach comes from successful relapse prevention in long-term trials of maintenance administration of intranasal esketamine, an S-enantiomer of racemic ketamine [50]. Similarly, administering continued ECT is a safe and effective way of reducing relapse rates following an acute course of ECT [51, 52]. Third, this trial involves the comprehensive collection of clinical, cognitive, neuroimaging, and molecular data through the CAN-BIND program [18, 21]. Such measures may elucidate the unique and overlapping mechanisms of action of ECT and ketamine, and, when added to the multiple data sources derived from other CAN-BIND trials, may contribute to identifying markers that predict treatment response and non-response to varied treatment strategies for MDEs. Such insight is critical for more tailored interventions in the future.

This trial is comparing the efficacy, speed of therapeutic effects, side effect profiles, and health care resource use associated with ketamine and ECT. As a non-inferiority trial, we expect ketamine to exhibit similar efficacy rates as ECT yet with more rapid antidepressant effects, less side effects, and with substantially less cost and health care resource use. This study is among the first to directly compare these treatments using a randomized design, complementing an ongoing, large-scale, parallel-arm clinical trial comparing ketamine and ECT for treatment-resistant depression in the United States (the ELEKT-D trial) [53]. Unique aspects of our trial include the crossover design, where participants who do not respond to the first randomized treatment may crossover to receive treatment with the alternate strategy, longer-term treatment and follow-up through the maintenance phase, and the inclusion of individuals with either unipolar or bipolar depression. Ketamine and ECT are both effective treatments for BP [54, 55], although, the assessment of effective and long-lasting treatments in the context of BP has received substantially less attention than that of unipolar depression.

Another strength of the current study is that both treatment arms are administered under comparable conditions. There is a balanced frequency of treatment administration in each arm during the acute treatment phases. Moreover, ECT and ketamine treatments are administered in the same setting at each site, reducing variability that could arise due to numerous confounding factors. Finally, since treatments cannot be blinded from participants or from clinical and research staff, clinical ratings are being conducted by raters who are blind to the treatment and are otherwise not involved in the trial. This rigorous study design allows for the relatively unbiased evaluation of the antidepressant effects of ketamine relative to an active comparator, addressing a critical gap in this area.

## Conclusion

With proper monitoring, ketamine may eventually be used as a less burdensome, potentially better tolerated, and less expensive alternative to ECT in severely depressed and/or treatment resistant patients experiencing a MDE. This clinical trial aims to directly compare the efficacy, speed of therapeutic effects, side effect profiles, and health care resource utilization associated with ketamine and ECT using a randomized, crossover design with well-articulated clinical endpoints. Further, it aims to examine the clinical utility of a novel maintenance strategy for ketamine. Finally, this trial may contribute to elucidating mechanisms underlying the antidepressant effects of ECT and ketamine, and identify predictors of treatment outcomes. Identifying biomarkers of response to ECT or ketamine could shorten delay to clinical improvement and increase remission rates. This study therefore has the potential to provide novel and significant progress in the treatment of MDEs in unipolar MDD and BP.

## Data Availability

Not applicable. This manuscript does not contain any data.

## Abbreviations

ATHF: Antidepressant Treatment History Form
BASE: BioArray Software Environment
BOLD: Blood oxygenation level-dependent
BP: bipolar disorder
Brain-CODE: Ontario Brain Institute Centre for Ontario Data Exploration
BSS: Beck Scale for Suicide Ideation
BW: Bandwidth
CAN-BIND: Canadian Biomarker Integration Network in Depression
CANMAT: Canadian network for mood and anxiety treatments
CGI: Clinical Global Impression
CNS: Central nervous system
CNS VS: CNS Vital Signs
C-SSRS: Columbia-Suicide Severity Rating Scale
DARS: Dimensional Anhedonia Rating Scale
DAST: Drug Abuse Screening Test
DNA: Deoxyribonucleic acid
DSMB: Data Safety and Monitoring Board
DSM-5: Diagnostic and Statistical Manual for Mental Disorders, fifth edition
DTI: Diffusion tensor imaging
ECT: Electroconvulsive therapy
EPI: Echo plan imaging
fBIRN: Functional Brain Imaging Research Network
FOV: Field of view
GAD: General Anxiety Disorder
Glx: Glutamate and glutamine
GRAPPA: Generalized autocalibrating partial parallel acquisition
Hz: hertz
IMHR: Royal’s Institute of Mental Health Research
IV: Intravenous
MADRS: Montgomery-Åsberg Depression Rating Scale
MDD: Major depressive disorder
MDE: Major depressive episode
ME-MPRAGE: Multi-echo magnetization prepared-rapid gradient echo
MINI: MINI International Neuropsychiatric Interview
mm: Millimetres
mMR: Molecular magnetic resonance
MOCA: Montreal Cognitive Assessment
MRI: Magnetic resonance imaging
mRNA: Messenger RNA
MRS: Magnetic resonance spectroscopy
ms: Milliseconds
NMDA: *N*-methyl-D-aspartate
OBI: Ontario Brain Institute
PE: Phase encoding [in MRI]
PET: Positron emission tomography
PHQ: Patient Health Questionnaire
PRESS: Point resolved spectroscopy
Q-LESQ-SF: Quality of Life Enjoyment and Satisfaction Questionnaire - short form
REDCap: Research Electronic Data Capture
RNA: Ribonucleic acid
rs-fMRI: Resting-state functional MRI
SF-36: Medical Outcome Study - short Form 36
SIGMA: Structured Interview Guide for the MADRS
SOP: Standard operating procedures
SPReD: Stroke Patient Recovery Research Database
T: Tesla
TE: Echo time [in MRI]
TR: Repetition time [in MRI]
TSES: Toronto Side Effect Scale
YMRS: Young Mania Rating Scale
